# Comparison of Ferric Sodium EDTA in Combination with Vitamin C, Folic Acid, Copper Gluconate, Zinc Gluconate, and Selenomethionine as Therapeutic Option for Chronic Kidney Disease Patients with Improvement in Inflammatory Status

**DOI:** 10.3390/nu14102116

**Published:** 2022-05-19

**Authors:** Antonella Giliberti, Annalisa Curcio, Nicola Marchitto, Luca Di Lullo, Fulvia Paolozzi, Fabiana Nano, Michele Pironti, Gianfranco Raimondi

**Affiliations:** 1 Department of Nephrology and Dialysis, “Santa Maria delle Grazie” Hospital Pozzuoli, 80078 Naples, Italy; gilaanto61@gmail.com; 2 Medical Department, Aqma Italia S.p.A., 80138 Naples, Italy; fabiana.nano@aqma.it (F.N.); michele.pironti@aqma.it (M.P.); 3 Department of Internal Medicine, “San Giovanni di Dio” Hospital, 04022 Fondi, Italy; n.marchitto@ausl.latina.it; 4 Department of Nephrology and Dialysis, “L. Parodi-Delfino” Hospital, 00034 Colleferro, Italy; dilulloluca69@gmail.com; 5 Department of Pharmacy, Pellegrini Hospital, 80134 Naples, Italy; fulvia.paolozzi@hotmail.com; 6 Department of Medico-Surgical Sciences and Biotechnologies, Faculty of Internal Medicine, “Sapienza” University of Rome, 04100 Latina, Italy; gianfrancoraimondi@uniroma.it

**Keywords:** chronic kidney disease, iron deficiency anemia, ferric sodium EDTA, ferritin, C-reactive protein, hepcidin, inflammation

## Abstract

Anemia is one of the most frequent and earliest complications of chronic kidney disease (CKD), which impacts a patient’s quality of life and increases the risk of adverse clinical outcomes. Patients’ inflammatory status is strictly related to the occurrence of functional iron deficiency anemia (IDA) because this causes an increase in hepcidin levels with the consequent inhibition of iron absorption and release from cellular stores into blood circulation. The aim of this study was to evaluate the use of the new oral formulation based on ferric sodium EDTA in combination with vitamin C, folic acid, copper gluconate, zinc gluconate, and selenomethionine (Ferachel Forte^®^) in patients with moderate CKD and functional IDA, analyzing the inflammatory status in addition to iron blood parameters, in comparison with oral ferrous sulfate and liposomal iron therapies. Sixty-two elderly patients were randomly allocated to one of the following oral treatments for 6 months: ferrous sulfate (Group 1; *N* = 20), ferric sodium EDTA in combination (Group 2; *N* = 22), and ferric liposomal formulation (Group 3; *N* = 20). The evaluated parameters included iron profile parameters of hemoglobin (Hb), sideremia, ferritin, transferrin saturation, C-reactive protein (CRP), and hepcidin. The results showed that in Group 1, there were no improvements. In Group 2, there were statistically significant (*p* < 0.001) improvements in all evaluated parameters. Finally, in Group 3, there were significant improvements in all evaluated parameters except for hepcidin, which was less than that of Group 2 patients. In conclusion, the findings showed the superior efficacy of the formulation based on ferric sodium EDTA over the other oral iron sources, and that this formulation can contribute to reducing the systemic inflammatory status in patients with CKD.

## 1. Introduction

Anemia is one of the most frequent and earliest complications of chronic kidney disease (CKD), which impacts a patient’s quality of life and increases the risk of adverse clinical outcomes, such as CKD progression, cardiovascular events, hospitalization, and all-cause mortality [1,2]. A recent review evaluated anemia prevalence in a U.S. CKD population and estimated an overall prevalence of anemia in 15.4% of CKD patients, with the percentage increasing with CKD stage to 17.4%, 50.3%, and 53.4% in stages 3, 4, and 5, respectively [1]. Another recent analysis conducted on over nine hundred thousand American veterans with non-dialysis-dependent CKD (NDD-CKD) showed an overall prevalence of anemia in 20.6% of patients, with a substantially higher percentage in patients at stage 3b (91.1%) and stage 4 CKD (96.1%) [3].

These relatively high percentages of patients with anemia and, in general, with iron deficiency (ID) in CKD patients are due to several pathophysiological mechanisms that range from the reduced renal production of erythropoietin to iron metabolism alterations, mainly caused by the chronic inflammatory status, to the negative martial balance due to the frequent gastrointestinal occult losses, the reduced intestinal iron absorption, and to the inadequate iron intake from the diet. For these reasons, two main forms of ID can be identified for CKD patients: absolute ID and functional ID, which, in case of high severity, can be classified as absolute iron deficiency anemia (IDA) and functional IDA [2,4].

Absolute IDA is characterized by a severe reduction in iron stores in the bone marrow, liver, and spleen, and can be defined as a reduction in all laboratory parameters used for measuring iron status (i.e., sideremia, hemoglobin (Hb), transferrin saturation, and ferritin). Functional IDA (also called anemia of chronic diseases) is characterized by the reduction in all parameters related to iron status, except for ferritin level, which is defined as >100 μg/L, indicating an increase in total body iron stores that that are unavailable for erythropoiesis. Functional IDA is mainly related to inflammatory status, which causes increased hepcidin levels [4,5]. Hepcidin is a hormone produced in the liver that regulates iron metabolism, binding, and the inducement of the internalization and degradation of ferroportin (FPN). FPN is an iron channel protein, present on the surface of enterocytes, macrophages, and hepatocytes, which allows iron absorption through the gut and iron release from cellular stores, where it is stored as ferritin, into blood circulation. During inflammation, hepcidin levels increase due to stimulation of its production in hepatocytes, which is mainly mediated by interleukin 6 (IL-6). Hepcidin inhibits FPN and the iron passage into the blood, resulting in iron deficiency [2,6].

Currently, the latest National Institute for Health and Care Excellence (NICE) guidelines recommend offering iron therapy to adults, children, and young people with anemia of CKD who are iron-deficient and who are not receiving ESA therapy. In particular, these guidelines specify the consideration of oral iron therapy before offering intravenous (IV) iron therapy in patients who are not receiving hemodialysis. Only if patients are intolerant to oral iron, or if target Hb levels are not reached within 3 months, is it recommended to switch to IV iron therapy [7]. In addition, in the KDIGO Clinical Practice Guideline for Anemia in Chronic Kidney Disease, it is acknowledged that both oral and IV administration routes of iron supplementation have advantages and disadvantages. Oral iron is cost-saving, easily available, and does not require IV access. However, traditional products in the market are often poorly tolerated and can have low iron absorption, causing adverse gastrointestinal events and limiting the efficacy of oral therapy. Additionally, efficacy and iron absorption are not concerns with IV iron, but it can expose patients to risk of adverse events regarding the injection site, hypersensitivity reactions, and iron overload, which can cause oxidative damage to the liver, heart, and kidneys and an increased incidence of infections, increased systemic inflammation, and decreased antioxidant defenses [8,9].

To overcome disadvantages related to poor tolerability and the lack of efficacy of traditional oral iron such as ferrous sulfate, an innovative oral iron formulation based on ferric sodium EDTA in combination with vitamin C, folic acid, copper gluconate, zinc gluconate, and selenomethionine (Ferachel forte^®^) was designed. Several studies demonstrated the safety and efficacy of this oral iron supplementation in different patient settings, such as the elderly, frailty, and with CKD from the low to moderate/severe stage [10,11,12,13,14,15].

Ferric sodium EDTA is a complex between ferric ion and ethylenediaminetetraacetate that provides a highly bioavailable and stable source of iron that is able to cross through the stomach without modification and to release ferric ion in the duodenal tract where it can be easily absorbed. Ferric sodium EDTA, as new iron source, showed broad efficacy and safety in several anemic patient types, such as pregnant women, anemic preschool- and school-aged children and adolescents [16,17,18,19,20,21,22,23,24,25,26]. All other active ingredients have been added to the formulation to improve iron absorption and promote erythropoiesis due to their synergistic effect. Vitamin C, copper, and zinc are known to positively influence iron absorption and promote the normal transport of iron into the body, whereas folic acid and selenium have an essential role in enzymatic processes, leading to hemoglobin production and DNA synthesis, thereby ensuring cellular viability [27,28,29].

Another oral iron source frequently used in patients with ID and/or IDA is liposomal iron, consisting of the salt ferric pyrophosphate, which is placed inside a liposome, a small vesicle 50–500 nm in diameter delimited by a phospholipid bilayer membrane that is similar to the cellular membrane. Liposome is absorbed through intestinal mucosa, and iron is released by liver enzymes, providing high gastrointestinal absorption and bioavailability [30].

Due to the involvement of inflammatory status in the regulation of hepcidin synthesis and, thus, in the occurrence of functional anemia, the evaluation of inflammatory parameters of CKD patients treated with oral iron therapy is of fundamental importance. For this reason, we conducted this preliminary study to evaluate the use of the new oral formulation based on ferric sodium EDTA in combination with vitamin C, folic acid, copper gluconate, zinc gluconate, and selenomethionine (Ferachel Forte^®^) in patients with moderate CKD and functional IDA, analyzing the inflammatory status in addition to the iron blood parameters of the patients, and compared the results with those of oral ferrous sulfate and liposomal iron therapies.

## 2. Materials and Methods

This was a multicenter, prospective, randomized, parallel-group, open-labeled study conducted on 62 patients (32 men and 30 women). Consecutive outpatients of 3 nephrology clinics in the Nephrology Department of Santa Maria delle Grazie Hospital of Pozzuoli (Naples), the Internal Medicine Department of San Giovanni di Dio Hospital of Fondi (Latina), and the Nephrology Department of L. Parodi-Delfino Hospital of Colleferro (Rome) were enrolled in the study.

Inclusion criteria were patients aged >18 years, with a diagnosis of functional IDA (with Hb concentration 10 < Hb < 13 g/dL in men and 10 < Hb < 12 g/dL in women, TSAT < 20% and ferritin level > 100 μg/L) with moderate CKD stage 3a–3b (30 < eGFR < 59 mL/min/1.73m^2^). Exclusion criteria were patients with anemia due to myelodysplasia, leukemia, or other oncological diseases; patients with severe CKD stage 4–5 and/or in hemodialysis; and patients under erythropoiesis stimulating agents (ESAs) therapy. Patients with severe CKD, and hemodialysis, and under ESA therapy were excluded to avoid any bias derived from the different therapies needed in these patient types.

All enrolled patients were randomly allocated to one of following oral treatment groups:

- Ferrous sulfate prolonged-release tablets (Ferro-grad^®^, Teofarma s.r.l., Pavia, Italy) 1 tab/day, containing 105 mg of ferrous ion for 6 months (Group 1; *N* = 20);
- Ferric sodium EDTA in combination with vitamin C, folic acid, copper gluconate, zinc gluconate, and selenomethionine (Ferachel Forte^®^, AQMA Italia S.p.A., Milan, Italy) 1 tab/day, containing 30 mg of ferric ion for 6 months (Group 2; *N* = 22);
- Ferric liposomal formulation (Ferroabi30^®^, Abi pharmaceutical s.r.l., Rome, Italy) 1 tab/day, containing 30 mg of ferric ion for 6 months (Group 3; *N* = 20).

A simple randomization was performed by using the Excel random function, matching every numerical ID patient to 1 of 3 treatment groups. The ID was assigned to every consecutive patient visiting the several departments involved in the study, who were enrolled in the study. The supplements were administrated every day in the morning.

Patients were evaluated for this study at basal condition (T0) and after 6 months of oral therapy (T1), performing laboratory tests to determine iron profile parameters of Hb, sideremia, ferritin, transferrin saturation (TSAT), and inflammatory parameter of high-sensitivity C-reactive protein (hs-CRP) along with hepcidin levels. Blood samples were collected in the morning after an overnight rest by antecubital venous puncture. Plasma and serum samples were obtained by centrifugation and frozen at −70 °C until further laboratory analysis. All blood parameters were measured using standard automated laboratory methods on a Cobas 6000 (Roche, Rotkreuz, Switzerland) using the relative kits, according to the manufacturer’s instructions.

The primary outcomes were the increase in Hb value and the reduction in inflammatory parameters at time T1, whereas secondary outcomes were the safety and tolerability of the several administered treatments during the study period. Nonadherence to therapy was defined as missed tablets more than 20% per month, and the check for this was performed by counting the number of tablets that remained in the box every month. Nonadherence was recorded on electronic medical records, and patients missing more than 20% of doses were excluded from the study. Adverse events that eventually occurred were recorded on electronic medical records, and resulted in the discontinuation of therapy due to adverse event onset.

Local ethic boards approved the study protocol. The study was conducted in accordance with the Declaration of Helsinki guidelines regarding ethical principles for medical research involving human subjects. All patients were included after receiving complete information regarding the study and after providing written informed consent.

Statistical analysis was performed using Paired T-test with Sigmastat v. 3.5 (San Jose, CA, USA) analysis program for Windows XP by comparing all blood parameters included in the study collected at T0 and T1. Data were normally distributed. Comparisons between groups were performed by one-way ANOVA Holm–Sidak test. The differences were considered significant when *p* < 0.05.

## 3. Results

**Figure 1:**
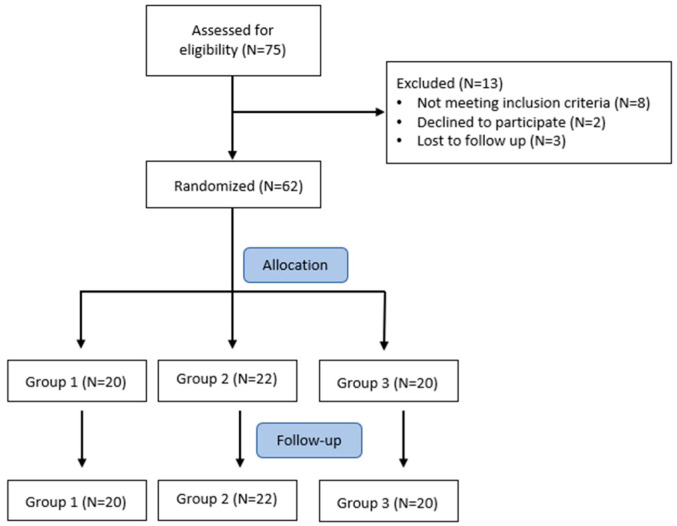
Flow diagram of patient selection in the study.

A total of 62 patients were included in the study. Figure 1 shows the flow diagram for the study. Enrolled patients were elderly (69.66 ± 5.11 years old) with a recent diagnosis of functional IDA (with Hb concentration < 13.0 g/dL in men and <12.0 g/dL in women, TSAT <20%, and ferritin level >100 μg/L) and with moderate CKD (eGFR 43.24 ± 7.30 mL/min/1.73 m^2^), not yet on ESA therapy. The baseline characteristics of the three groups of patients are shown in Table 1.

The blood parameters evaluated in the three groups of patients treated with ferrous sulfate (Group 1); ferric sodium EDTA in combination with vitamin C, folic acid, copper gluconate, zinc gluconate and selenomethionine (Group 2); or ferric liposomal formulation (Group 3) are reported in Table 2.

Group 1 did not show statistically significant changes in any of the evaluated parameters, except for ferritin, which increased, indicating a worsening in functional IDA. Group 2 showed a statistically significant (*p* < 0.001) improvement in all evaluated parameters. In particular, the Hb value increased on average by 1.21 g/dL, the sideremia value increased on average by 25.41 μg/dL, the TSAT value increased on average by 16.77%, and ferritin value decreased on average by 84.95 μg/L, showing an improvement of functional IDA. Furthermore, the inflammatory parameters of CRP in Group 2 patients showed a significant reduction, on average by 2.53 mg/dL, along with the hepcidin value decreasing on average by 9.95 ng/mL. Group 3 patients experienced significant improvements in all evaluated parameters except for hepcidin value; however, the changes were inferior to those shown by Group 2 patients (*p* < 0.001).

For statistical analysis between groups, a one-way ANOVA Holm–Sidak test was performed, showing a significant difference for all parameters evaluated at T1 (*p* < 0.001), confirming the superior efficacy of the Group 2 treatment.

The renal function in each group of patients did not change between T0 and T1 because, at the end of study eGFR values were 46.10 (±1.92) mL/min/1.73 m^2^ in Group 1, 35.32 (±7.49) mL/min/1.73 m^2^ in Group 2, and 47.55 (±2.04) mL/min/1.73 m^2^ in Group 3 (*p* < 0.001 for all groups).

Treatment with ferric sodium EDTA combination and/or ferric liposomal formulation were safe and well-tolerated because patients in Groups 2 and 3 did not report any adverse events (AEs) during treatment. Instead, 35% of Group 1 patients (*N* = 7) reported gastrointestinal (GI) AEs, mainly constipation, diarrhea, nausea, abdominal cramps, and vomiting. No patient discontinued therapy.

## 4. Discussion

This was a preliminary comparative study conducted on 62 patients with functional IDA and moderate CKD, randomized to treatment with three different iron sources for oral administration, where, in addition to the iron blood parameters used to demonstrate treatment efficacy, CRP and hepcidin values were evaluated to deepen our understanding of the effect of therapy on inflammatory status and its involvement in iron homeostasis, which is strictly related to hepcidin levels in the body.

In patients with CKD, a frequent complication is functional IDA, which is strongly influenced by the inflammatory status, causing increased levels of hepcidin. Several studies confirmed the involvement of hepcidin as a major key factor for iron homeostasis because it binds and inhibits ferroportin (FPN). FPN is the membrane transporter that allows iron to exit from the cells (enterocytes, hepatocytes, and macrophages) to enter the blood stream. Hepcidin production is upregulated by proinflammatory cytokines such as IL-1β and IL-6. High levels of hepcidin cause major FPN inhibition, which implies a sequestration of iron in the cell in the form of ferritin and the occurrence of functional IDA. A common inflammatory marker is CRP, which is positively correlated with ferritin levels. For this reason, in this study, we also evaluated the effect of several treatments on CRP and hepcidin levels, in addition to iron parameters [2,6,31].

The efficacy results (Table 2) clearly show that the best improvement occurred within the Group 2 patients, who were treated with ferric sodium EDTA in combination with vitamin C, folic acid, copper gluconate, zinc gluconate, and selenomethionine (Ferachel Forte^®^). These patients had the highest values of Hb, sideremia, and TSAT, while the ferritin value significantly decreased, showing a trend of normalization toward this value because the iron stored in the cells became more available for the needs of the organism and for erythropoiesis. Furthermore, in Group 2 patients, the CRP results were statistically significantly lower compared with the baseline, showing very high relevance with respect to the other treatment groups. Similar results were found for hepcidin, which was significantly reduced in Group 2 patients, but did not occur in the other two treatment groups.

This effect allowed us to hypothesize that ferric sodium EDTA in combination with vitamin C, folic acid, copper gluconate, zinc gluconate, and selenomethionine (Ferachel Forte^®^) therapy resulted in an improvement in the inflammatory status in patients evaluated, which was not found in the patients treated with the other iron sources.

Ferric sodium EDTA is a novel source of iron previously used in numerous studies with important results regarding efficacy and safety in different patient types, such as pregnant women, anemic preschool- and school-aged children, and adolescents [16,17,18,19,20,21,22,23,24,25,26]. This iron source has received approval from major regulatory authorities, such as the WHO, FDA, and EFSA, as an iron source with elevated bioavailability and a good safety profile [32,33,34].

The formulation of ferric sodium EDTA with vitamin C, folic acid, copper gluconate, zinc gluconate, and selenomethionine (Ferachel Forte^®^), which was designed to improve iron absorption and provide several nutrients required for erythropoiesis, showed encouraging efficacy and safety results in previous studies in several anemic patient types, including the elderly, frailty, and patients with CKD from low to moderate/severe stage [10,11,12,13,14,15].

According to the latest NICE guidelines, trial oral iron therapy is recommended before offering IV iron therapy in patients who are not on ESA therapy and not receiving hemodialysis. We consider that this new oral iron source, based on the association of ferric sodium EDTA with other important nutrients, represents a solution to avoid IV iron therapy, which is recommended when patients are intolerant to oral iron or did not reach target Hb levels within 3 months, because it is well-tolerated and produces significant efficacy results [7].

Notably, treating IDA in patients with CKD with oral therapies is a way to avoid the risks related to IV iron administration, including severe allergic reactions; adverse reactions at the injection site such as pain, swelling, phlebitis, and thrombophlebitis; and iron overload risk, which can cause oxidative damage to the liver, heart, and kidneys and an increased incidence of infections, increased systemic inflammation, and decreased antioxidant defenses [35]. In relation to limiting the risk of severe allergic reactions and anaphylactic shock related to IV iron administration, the latest European Medicines Agency (EMA) recommendations state that IV iron medicines should only be administered when staff trained to evaluate and manage anaphylactic and anaphylactoid reactions are immediately available as well as resuscitation facilities [36]. These indications make IV iron administration harder to handle and more expensive for national health systems and the patients.

Treatment with ferric sodium EDTA in combination with vitamin C, folic acid, copper gluconate, zinc gluconate, and selenomethionine (Ferachel Forte^®^) can represent a safe and effective therapeutic alternative to conventional oral and IV iron therapy, showing better results than the other oral therapies considered in this study. Furthermore, the results regarding CRP and hepcidin values are of particular interest because they allowed us to hypothesize that the treatment with ferric sodium EDTA can normalize patients’ inflammatory status, making iron, stored as ferritin, more usable for erythropoiesis and decreasing the iron sequestration that occurs when there are high levels of hepcidin in the blood circulation.

The results of this study are interesting, but due to the small sample size, we cannot generalize these effects to the CKD population. Furthermore, larger studies are necessary both in CKD patients with functional IDA and in patients with other inflammatory conditions, which are also not kidney-related, such as anemic patients with chronic inflammatory bowel diseases and with onco-hematological pathologies.

In conclusion, this preliminary study provided interesting results regarding the superior efficacy of treatment with ferric sodium EDTA in combination with vitamin C, folic acid, copper gluconate, zinc gluconate, and selenomethionine (Ferachel Forte^®^) compared with the other evaluated oral iron sources, both in relation to the possibility that the treatment with this new oral iron product, in addition to a significant improvement in iron blood parameters, can contribute to reducing the systemic inflammatory status in patients with CKD.

## Figures and Tables

**Table 1:** Baseline characteristics of Group 1 (treated with ferrous sulfate), Group 2 (treated with ferric sodium EDTA in combination with vitamin C, folic acid, copper gluconate, zinc gluconate, and selenomethionine), and Group 3 (treated with liposomal iron).

Variable	Group 1 (*N* = 20)	Group 2 (*N* = 22)	Group 3 (*N* = 20)	*p* Value *
Male/female, N	10/10	11/11	11/9	-
Age (years), mean (±SD)	68.3 (2.8)	72.3 (7.5)	68.05 (1.7)	-
eGFR (mL/min/1.73 m^2^),	46.15 (2.2)	36.45 (8.5)	47.80 (3.5)	<0.001
Hb (g/dL), mean (±SD)	10.46 (0.22)	10.70 (0.23)	10.59 (0.14)	0.023
Sideremia (μg/dL), mean (±SD)	28.15 (1.96)	38.18 (7.17)	27.10 (1.87)	0.004
TSAT (%), mean (±SD)	16.85 (3.15)	19.95 (3.07)	17.45 (4.32)	0.041
Ferritin (μg/L), mean (±SD)	279.10 (33.53)	249.68 (37.50)	269.45 (56.94)	0.170
CRP (mg/dL), mean (±SD)	4.56 (0.30)	4.05 (0.36)	4.63 (0.15)	0.001
Hepcidin (ng/mL), mean (±SD)	19.01 (0.39)	18.85 (0.44)	18.92 (0.48)	0.442

* One-way ANOVA Holm–Sidak test.

**Table 2:** Blood parameters of patients treated with ferrous sulfate (Group 1); ferric sodium EDTA in combination with vitamin C, folic acid, copper gluconate, zinc gluconate, and selenomethionine (Group 2); or ferric liposomal formulation (Group 3).

Blood Parameters, Mean (±SD)	Group (*N* = 20)			Group 2 (*N* = 22)			Group 3 (*N* = 20)		
	T0	T1	*p* Value	T0	T1	*p* Value	T0	T1	*p* Value
**Hb (g/dL)**	10.46 (0.22)	10.42 (0.19)	0.780	10.70 (0.23)	11.91 (0.21)	**<0.001**	10.59 (0.14)	10.78 (0.22)	**<0.001**
**Sideremia (μg/dL)**	28.15 (1.96)	30.65 (4.07)	0.117	38.18 (7.17)	63.59 (8.28)	**<0.001**	27.10 (1.87)	33.05 (3.26)	**<0.001**
**TSAT (%)**	16.85 (3.15)	17.11 (4.51)	0.874	19.95 (3.07)	36.72 (4.28)	**<0.001**	17.45 (4.32)	19.60 (2.73)	**<0.001**
**Ferritin (μg/L)**	279.10 (33.53)	291.75 (38.17)	**<0.001**	249.68 (37.50)	164.73 (28.73)	**<0.001**	269.45 (56.94)	217.70 45.86)	**<0.001**
**CRP (mg/dL)**	4.56 (0.30)	4.53 (0.21)	0.576	4.05 (0.36)	1.52 (0.32)	**<0.001**	4.63 (0.15)	4.06 (0.63)	**0.012**
**Hepcidin (ng/mL)**	19.01 (0.39)	18.85 (0.44)	0.673	18.85 (0.44)	8.90 (1.18)	**<0.001**	18.92 (0.48)	18.62 (0.63)	0.245
